# Asymmetric Dispersal and Colonization Success of Amazonian Plant-Ants Queens

**DOI:** 10.1371/journal.pone.0022937

**Published:** 2011-08-03

**Authors:** Emilio M. Bruna, Thiago J. Izzo, Brian D. Inouye, Maria Uriarte, Heraldo L. Vasconcelos

**Affiliations:** 1 Department of Wildlife Ecology and Conservation and Center for Latin American Studies, University of Florida, Gainesville, Florida, United States of America; 2 Departamento de Botânica e Ecologia, Universidade Federal de Mato Grosso, Cuiabá, Mato Grosso, Brazil; 3 Department of Biological Science, Florida State University, Tallahassee, Florida, United States of America; 4 Department of Ecology, Evolution and Environmental Biology, Columbia University, New York, New York, United States of America; 5 Instituto de Biologia, Universidade Federal de Uberlândia, Uberlândia, Minas Gerais, Brazil; 6 Biological Dynamics of Forest Fragments Project, Instituto Nacional de Pesquisas da Amazônia & Smithsonian Tropical Research Institute, Manaus, Amazonas, Brazil; University of California, Berkeley, United States of America

## Abstract

**Background:**

The dispersal ability of queens is central to understanding ant life-history evolution, and plays a fundamental role in ant population and community dynamics, the maintenance of genetic diversity, and the spread of invasive ants. In tropical ecosystems, species from over 40 genera of ants establish colonies in the stems, hollow thorns, or leaf pouches of specialized plants. However, little is known about the relative dispersal ability of queens competing for access to the same host plants.

**Methodology/Principal Findings:**

We used empirical data and inverse modeling—a technique developed by plant ecologists to model seed dispersal—to quantify and compare the dispersal kernels of queens from three Amazonian ant species that compete for access to host-plants. We found that the modal colonization distance of queens varied 8-fold, with the generalist ant species (*Crematogaster laevis*) having a greater modal distance than two specialists (*Pheidole minutula*, *Azteca* sp.) that use the same host-plants. However, our results also suggest that queens of *Azteca* sp. have maximal distances that are four-sixteen times greater than those of its competitors.

**Conclusions/Significance:**

We found large differences between ant species in both the modal and maximal distance ant queens disperse to find vacant seedlings used to found new colonies. These differences could result from interspecific differences in queen body size, and hence wing musculature, or because queens differ in their ability to identify potential host plants while in flight. Our results provide support for one of the necessary conditions underlying several of the hypothesized mechanisms promoting coexistence in tropical plant-ants. They also suggest that for some ant species limited dispersal capability could pose a significant barrier to the rescue of populations in isolated forest fragments. Finally, we demonstrate that inverse models parameterized with field data are an excellent means of quantifying the dispersal of ant queens.

## Introduction

The approximately 14,000 species of ants (family Formicidae) account for roughly one-third of the world's insect biomass [1]. The dispersal ability of foundress queens is central to understanding ant life-history evolution, and also plays a fundamental role in ant population and community dynamics, the maintenance of genetic diversity, and the spread of invasive ant species [2], [3], [4], [5]. However, with the exception of a few well-studied species [5], [6], little is known regarding the distances queens typically disperse when they leave their colonies to found new nests or the maximum distances they are capable of dispersing (but see e.g., [4], [7], [8]). This is because techniques commonly used to track dispersing animals (e.g., mark-recapture methods, radio transmitters) are rarely applicable to ants given their size, the structural complexity of the habitat through which they disperse, and the difficulty in identifying and surveying all potential nesting sites. Though genetic techniques for estimating dispersal appear promising [4], [8], their application may be limited owing to their stringent assumptions and challenges in sampling intensively enough to accurately estimate dispersal.

The dominance of ants is particularly pronounced in the tropics, where in addition to their numerical superiority they are critical predators, herbivores, ecosystem engineers, and agricultural pests [1]. Species from at least 40 genera of tropical ants also establish colonies in the specialized stems, hollow thorns, leaf pouches, or petioles of plants known as ‘myrmecophyes’; these ants defend host-plants against herbivores and prune encroaching vegetation [9], [10]. Multiple ant species often vie for the same species of host-plant [11], [12], and vacant plants in which queens can establish colonies are a limiting resource for which there is intense competition [13], [14], [15]. Theory suggests that interspecific differences in the dispersal capability of ant queens play a key role in the maintenance of diversity in these communities, either via tradeoffs between dispersal ability and other life-history traits (e.g., competitive ability, colony fecundity), or from the interaction of dispersal limitation with spatial heterogeneity in host-plant density (reviewed in [3]). Studies in multiple plant-ant systems have demonstrated inequities in the competitive ability of ant queens or colonies [16], [17], plant and colony distribution consistent with habitat partitioning and patch dynamics [18], [19], and patterns of colonization that imply dispersal limitation [8], [12], [20] or interspecific variation in dispersal ability [20], [21]. Nevertheless, drawing general conclusions regarding the importance of dispersal for plant-ant coexistence requires quantitative descriptions of dispersal for multiple ants competing for access to the same host-plants.

**Figure 1:**
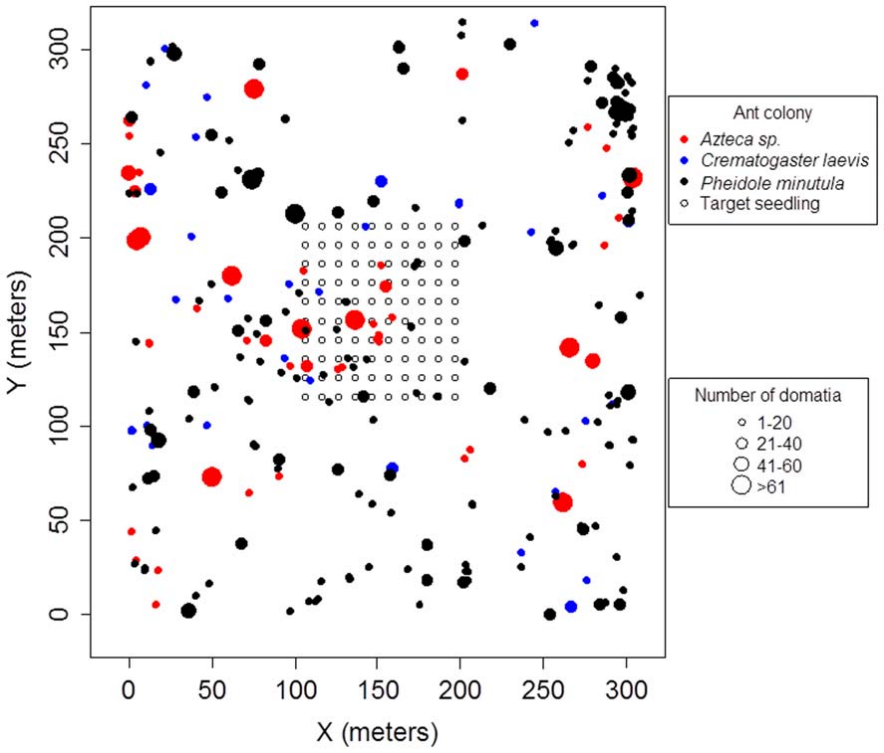
Map of established ant colonies and target seedlings. Location and size of plants hosting colonies of *Azteca* sp., *Crematogaster laevis*, and *Pheidole minutula* and the location of experimentally planted seedlings (“trap plants”) of *Maieta guianensis* and *Tococa bullifera*.

**Figure 2:**
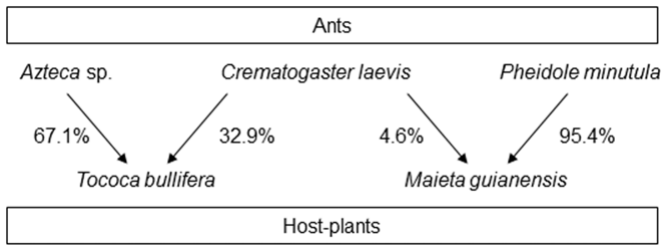
Focal community of ant-plant mutualists. Graphical depiction of the Amazonian plant-ant community used to quantify dispersal capability of ant queens. Values by arrows are the percentage of host-plants colonized by each species of ant in our 9-ha study site.

The biology of myrmecophytes provides a unique opportunity to circumvent the challenges associated with quantifying ant queen dispersal in other systems. The ant species that nest in these plants do so obligately, and each is associated with a limited subset of plant species [11]. Consequently, all ant colonies in a site, as well as all nesting sites to which queens could potentially disperse, can be readily identified by mapping the distribution of host plants [8], [22]. We mapped all individuals of the understory shrubs *Maieta guianensis* and *Tococa bullifera* (both Melastomataceae) in 9 hectares of primary forest in the central Amazon (Figure 1). These two plant species serve as hosts for three species of ant symbionts that nest exclusively in their domatia: *Crematogaster laevis*, *Pheidole minutula*, and an undescribed species of *Azteca* [11], [18], [22]. *Crematogaster laevis* competes for access to host plants with both *Azteca* sp. and *P. minutula* (Figure 2), and it has been hypothesized [21] that superior dispersal ability promotes its persistence in this system despite the inferior competitive ability of queens competing for access to host-plant seedlings [16], the poor defense colonies provides host-plants against herbivores [23], its low rates of colony persistence [18], and the high mortality rates of the host plants it occupies [18]. After mapping all colonies of the three ant species, we transplanted vacant, greenhouse-grown seedlings of their host plants (N = 50 individuals of each species) into the central hectare of the plot and repeatedly surveyed them for colonization by ant queens (see Materials and Methods). These data, coupled with the location and size of established colonies, allowed us to estimate a probability density function describing the spatial redistribution of successfully dispersing queens (i.e., the ‘dispersal kernel’) of each ant species using ‘inverse modeling’ – a technique developed by plant ecologists to estimate the distances seeds are dispersed from fruiting trees [24], [25], [26]. To our knowledge this is the first application of inverse modeling techniques to calculate the dispersal kernels of animals.

## Results

**Figure 3:**
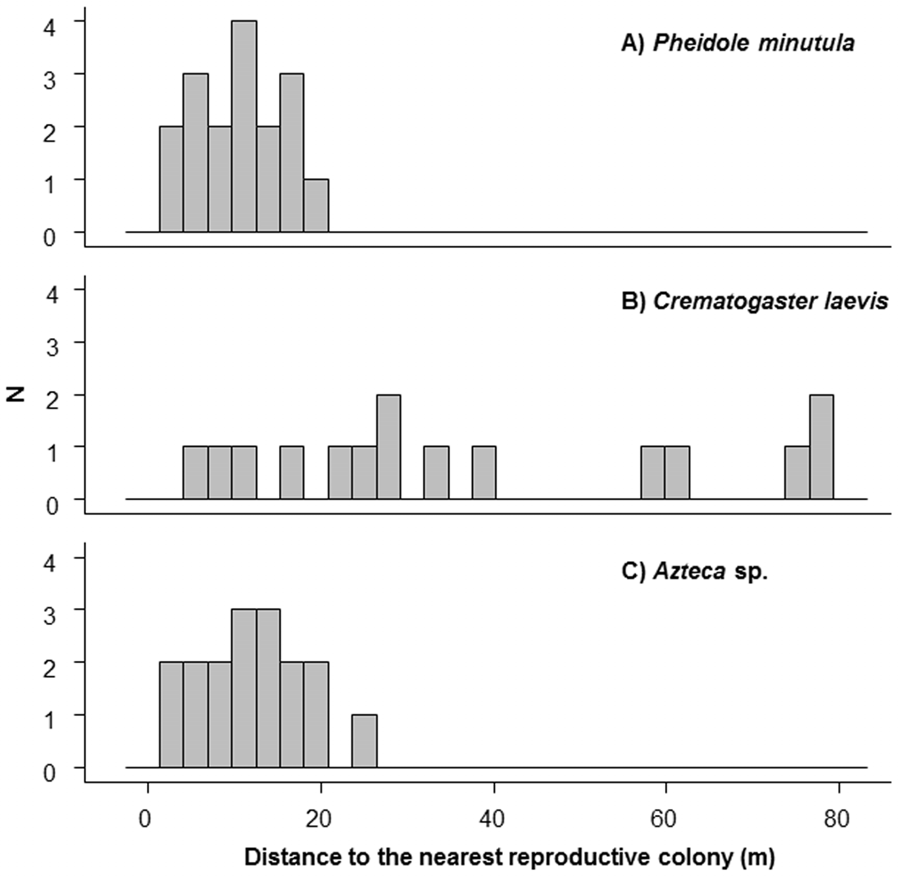
Distance from colonized seedlings to the nearest reproductive ant colony. Histograms of the pairwise distances from each colonized trap plant to the nearest reproductive colony of the ant species that colonized it. A) *Pheidole minutula*: mean pairwise distance = 10.91 m±5.26 SD, B) *Crematogaster laevis*: mean pairwise distance = 37.49 m±25.92 SD, C) *Azteca* sp.: mean pairwise distance = 12.30 m±6.53 SD.

**Figure 4:**
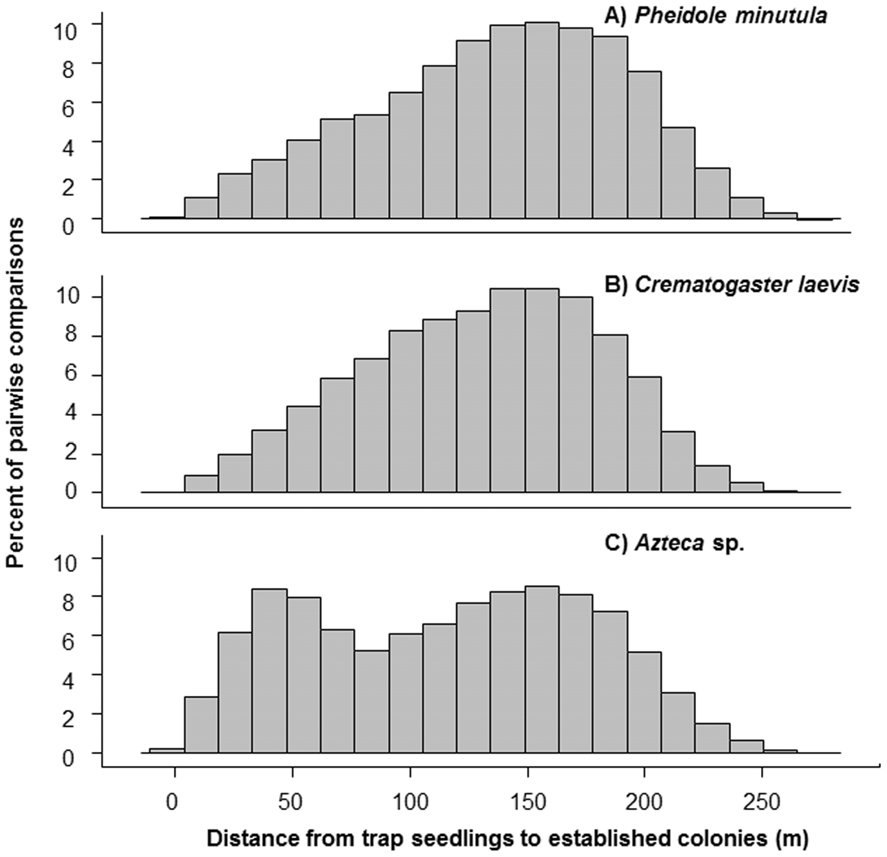
Pairwise distances from established colonies to trap plants. Histograms of the distance from trap plants to colonies for each of the three focal ant species. The X axis shows the percentage of all colony-trap pairwise comparisons. A) *Pheidole minutula*: mean colony-trap distance = 137.59 m±53.69 SD, B) *Crematogaster laevis*: mean colony-trap distance = 130.88 m±60.60 SD, C) *Azteca* sp.: mean colony-trap distance = 116.09 m±59.84 SD.

**Figure 5:**
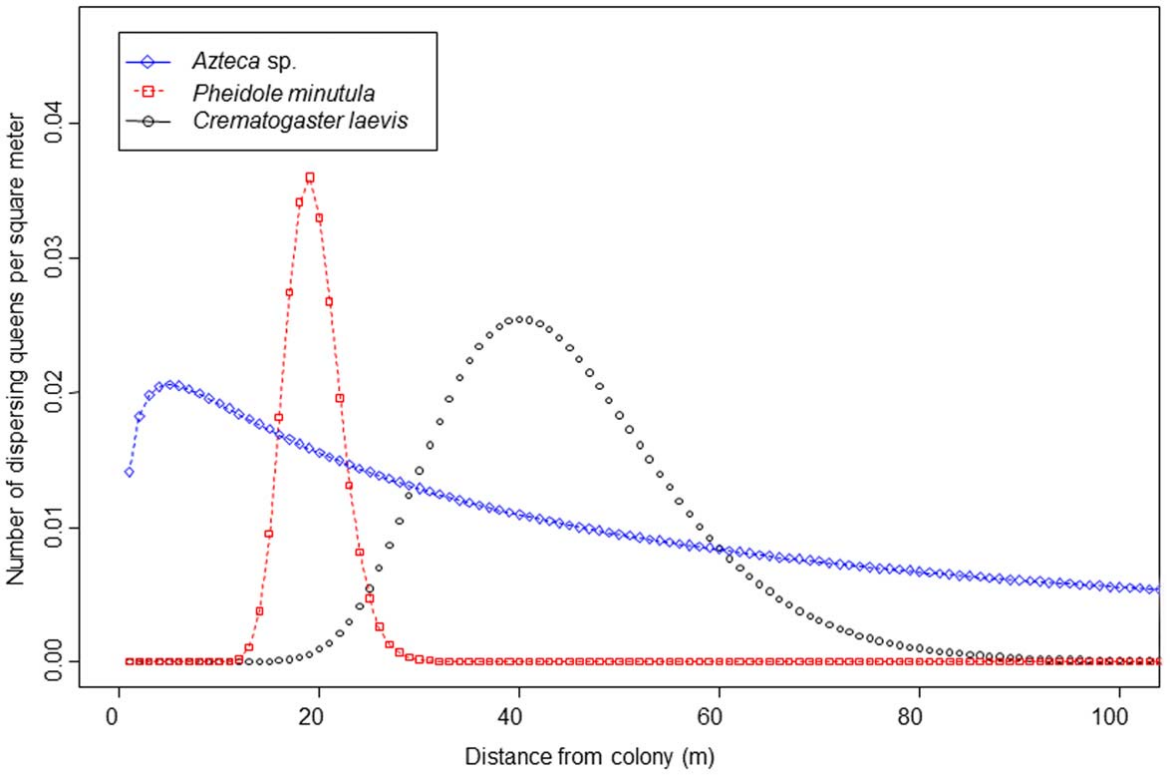
Dispersal kernels for three species of Amazonian plant-ants. Dispersal kernels (i.e., probability density functions describing the spatial redistribution of queens around reproductive colonies) for ant queens obligately nesting in *Tococa bullifera* or *Maieta guianensis*. These kernels are scaled for a colony housed in a plant of the median size observed in our 9-ha study plot.

The median distance from colonized seedlings to the nearest potentially reproductive colony was significantly different among ant species (Kruskal-Wallis, H = 13.96, df = 2, *p*<0.001, Figure 3); experimentally transplanted vacant seedlings (hereafter, “trap plants”, analogous to seed traps used in plant ecology) colonized by *Crematogatser laevis* queens were significantly further from reproductive colonies than those trap plants colonized by queens of either *Azteca* sp. or *Pheidole minutula* (Steele's Nonparametric Multiple Comparison Test [27], Table 1). However, this is not because established *C. laevis* colonies were located further from trap plants. There was a significant difference among colonies of the different species in their proximity to trap plants (Table 2), but *P. minutula* colonies were actually further from trap plants than those of *C. laevis* (average distances from trap plants to colonies: *Azteca* sp.: 116.09 m±59.84 SD, *C. laevis*: 130.88 m±60.60 SD, *P. minutula*: 137.59 m±53.69 SD, Figure 4). Instead, our inverse models suggest *C. laevis* queens establish colonies furthest from natal colonies. Assuming a log-normal kernel (see Materials and Methods), the modal dispersal and colonization distance of *Crematogaster laevis* queens is double that of *Pheidole minutula* queens (40.1 m and 18.9 m, respectively) and eight-fold that of *Azteca* sp. (5 m; Table 3, Figure 5). The kernels also had very different shapes (Figure 5), suggesting that the maximal colonization distance of *C. laevis* is approximately 80 m, while queens of *Azteca* sp. may be capable of infrequent movements in excess of 400 m.

**Table 1:** Result of Steel's Test comparing the median distance of colonized trap plants to the nearest reproductive colony for all pairwise comparisons of ant species.

Comparison	Relative Effect, 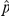 (lower-upper 95% confidence limits)	*p* value
*Azteca* sp. vs. *Crematogaster laevis*	0.82 (0.58–1.07)	0.005
*Azteca* sp. vs. *Pheidole minutula*	0.45 (0.21–0.68)	0.87
*C. laevis* vs. *P. minutula*	0.15 (−0.09–0.40)	0.002

**Table 2:** Nested Analysis of Variance comparing the average distance of trap plants to colonies of the three ant species (*Crematogaster laevis*, *Azteca* sp., *Pheidole minutula*) mapped in our 9 ha study site.

Source	df	MS	F	*P*
Ant Species	2	496230	169.68	<0.0001
Trap (Ant Species)	3	74	0.025	0.99
Error	16694	48822953		

(Nested ANOVA; Main effect of Ant species: F_2,16694_ = 169.68, P = <0.0001).

**Table 3:** Maximum-likelihood parameter estimates (MLE) and 95% support intervals (SI) for inverse models estimating the dispersal kernels of three mutualist ant species nesting in two species of Amazonian ant-plants.

	*Pheidole minutula*	*Azteca* sp.	*Crematogaster laevis*
Parameter 1	MLE (lower SI-Upper SI)	MLE (lower SI-Upper SI)	MLE (lower SI-Upper SI)
*X_0_*	18.85 (17.42–20.47)	5 (5–5.05)	40.16 (31.54–52.16)
*X_b_*	0.14 (0.09–0.23)	1.85 (1.21–1.93)	0.27 (0.15–0.66)
*a*	1.58 (1.00–4.00)	11.40 (7.45–22.57)	14.84 (8.75–23.36)
*b*	55.07 (36.50–96.20)	114.15 (−10.00–250.00)	1.91 (0–10)

1 Parameters: *X_0_* = Mode of the log-normal dispersal kernel, *X_b_* = Variance of the log-normal dispersal kernel, *a* = Slope of the line describing the relationship between plant size and queen production, *b* = Intercept of the relationship between plant size and queen production.

## Discussion

We were able to estimate the shapes of effective dispersal kernals for queens of three ant species. The dispersal kernel for *Crematogaster laevis* had the greatest mode, suggesting that it generally disperses further than either ant species with which it competes for access to host plants. However, our results also revealed the potential for long-distance dispersal events by *Azteca* sp. That potential partner ant species differ significantly in their capacity to disperse to and colonize host-plants plants may help explain patterns of colonization and ant colony distribution previously observed in this [18] and other [19], [28], [29] ant-plant systems. Along with the lack of specialized entrances to domatia (i.e., “lock-and-key” mechanisms, sensu [30]), interspecific differences in dispersal and colonization success may also be important mechanisms inhibiting the evolution of further specialization in ant-plant systems, in which there are often large differences in the quality of defense and host plant fitness associated with different ant partner species [18], [23], [31], [32].

It has previously been suggested [21] that smaller body size, and hence flight muscles, may explain why the *Pheidole minutula* queens have lower dispersal distances than those of *Crematogaster laevis*; the same appears to be true in other ant-plant systems [20]. However, the *Azteca* sp. queens have the lowest modal dispersal distance of these three species, despite being similar in size to *C. laevis*. Given the potential for long distance dispersal by queens of *Azteca* sp., we hypothesize that this shorter modal dispersal distance instead reflects their superior efficiency at finding host plants. Testing this hypothesis will be challenging – it remains a mystery how plant-ant queens in flight identify host-plant seedlings against a backdrop of hundreds of other plant species [9], [33]. However, it is likely they use a combination of visual and olfactory cues, as is the case with phytophagous insects [34]. Indeed at short distances, queens have been shown to use volatiles emitted by plants to discriminate host-plants from closely related but non-myrmecophytic species [33], [35], [36]. It may be that *Azteca* sp. queens have the ability to detect these volatiles at greater distances than their competitors, superior abilities to identify the shape of plants and the characteristic venation patterns of host-plant leaves, or both.

It is notable that the modal ant queen dispersal distances we estimated with inverse models are shorter than the average distances inferred using other techniques [4], [8], [20] and well below the potential dispersal capacity suggested by observations of ants in novel or experimentally created habitat patches [5], [7]. If host plant density is greater in our sites than in other systems, then queens might only be required to disperse short distances to find vacant host plants. A more likely explanation, however, is that previous studies have overestimated dispersal. This could result from not exhaustively mapping all potential source and destination host-plants in a site [8], [20], thereby missing many short-distance dispersal events.

Our study has two important caveats. First, it was conducted entirely during a three month period during the dry season. Little is known regarding the environmental cues that stimulate the nuptial flights of ant queens in tropical forests [37], but the colonization of seedlings by *Pheidole minutula* in our field sites appears to be closely linked to precipitation ([21]; see also [37] for evidence from Peru of similar seasonality in colonization of *Cordia* by *Allomerus octoarticulatus*). If this seasonal variation in host plant colonization by *P. minutula* is common, then caution should be taken in estimating the total number of colonizations per year using our data. Second, we could be overestimating dispersal distances for all three species if queens arrive at experimental seedlings but left without attempting to colonize them or died prior to entering domatia. The low density of vacant plants [15], [22] probably makes it extremely costly for a queen to disperse again once she has arrived at a host-plant seedling, and extensive field observations indicate that upon arriving at a seedling queens of all three focal taxa immediately shed their wings and attempt to enter domatia (HLV and TJI, *personal observation*). Some queens will probably die prior to colonizing the seedling on which they land, however, and there is some experimental evidence that *P. minutula* successfully enters domatia at a higher rate than *C. laevis* [16]. It is therefore possible that using colonization of trap-plants by queens as a proxy for dispersal means that our results are conservative estimates true dispersal ability, especially for *C. laevis*. If so, our estimates of dispersal might best be called ‘realized dispersal’, i.e., dispersal followed by successful colonization [8], [20].

In conclusion, our results have implications for the study of plant-ant diversity in tropical ecosystems. First, tropical forests are increasingly fragmented by human activities, which isolates populations of ant-plant partners [22]. The mating system of social insects makes them particularly susceptible to inbreeding [38], and isolated populations are frequently smaller than those in unbroken forest [22]. If the distance separating fragments proves a barrier to dispersal for queens of some species, this will increase the likelihood that isolated populations of ants and their host-plants could suffer the detrimental effects of demographic, environmental, or genetic stochasticity [8]. Second, a critical but rarely documented requirement of some mechanisms that promote coexistence in ant-plant communities is that poorer competitors or habitat specialists are superior dispersers. Our results are consistent with this hypothesis, but also suggest that attempting to categorize species as “good” or “poor” dispersers when testing models of competition-colonization tradeoffs is overly simplistic – is the best disperser the one that has the greatest potential dispersal distance or the one that dispersers further on average? Finally, we show that an inverse modeling approach can help overcome the challenges in quantifying ant dispersal in structurally complex habitats, not the least of which is the difficulty in documenting rare long-distance dispersal events [26].

## Materials and Methods

### Ethics Statement

All research was conducted with the approval of Brazil's National Council of Scientific and Technological Development (CNPq, Permit Number 276/2005) and the Brazilian Institute of Environment and Renewable Natural Resources (IBAMA, Permit Number 226/2005).

### Field Site and data collection

Fieldwork was conducted January–September 2007 in Reserve #1501 of the Biological Dynamics of Forest Fragments Project (BDFFP). This 1,000 ha reserve is located 70 km north of Manaus, Brazil (2°30′S, 60°W) and is embedded in a large (>10,000 ha) expanse of primary forest. The habitat is non-flooded lowland rain forest, with a 30–35 m tall canopy and an understory dominated by stemless palms. Soils in the sites are highly acidic and nutrient poor xanthic farralsols with poor water retention capacity [39]. Annual rainfall ranges from 1,900–3,500 mm per year, and there is a pronounced dry season from June–October [40].

*Tococa bullifera* (Melastomataceae) is an understory shrub that grows to a maximum height of 2–3 m. It has two pouches at the base of each leaf in which ant queens establish colonies [18], [41]. *Maieta guianensis* (Melastomataceae), also an understory shrub, grows to a height of 1.5 m [18], [21]. It has highly dimorphic paired leaves, with a pair of foliar pouches at the base of the larger leaves in which ants nest. In our study sites two ant species are associated with *M. guianensis*; most plants contain colonies of *Pheidole minutula* (95%), with the remainder occupied by *Crematogaster laevis* (5%). The ant associates of *T. bullifera* are an undescribed species of *Azteca* (67%) and *Crematogaster laevis* (∼33%) (Figure 2). These frequencies are similar to those reported in previous surveys conducted in our field sites [18]. Although a previous study conducted in our study sites [11] has treated the *Azteca* species that colonizes *T. bullifera* and *M. guianensis* as the same one colonizing the sympatric myrmecophyte *Cordia nodosa* (Boraginaceae), this appears to be a misidentification resulting from the use of worker morphology to differentiate species. The complex taxonomy of *Azteca* requires using queens to distinguish species [42]; differences between *Azteca* queens from *C. nodosa* and those from *T. bullifera* in size, coloration, the shape of the propodeum, and the number of propodeal hairs strongly suggest these are distinct species (T. Izzo, *unpubl. data*). Although seedlings of both plant species can harbor incipient (i.e., non-reproductive) colonies of more than one ant species, adult plants house just a single colony of only one species. In addition to scavenging for insects on the leaf surface, resident ants tend coccids for honeydew inside domatia [43], [44].

From January–July 2007 we demarcated a 9-ha plot at reserve 1501 and then marked and mapped all *Maieta guianensis* and *Tococa bullifera* in the plot (Figure 1). For each plant we recorded the identity of its ant resident estimated its size by counting the number of domatia bearing leaves. We mapped a total of 217 *M. guianensis* (*n* = 10 with *Crematogaster laevis* colonies, *n* = 207 with *Pheidole minutula* colonies) and 79 *Tococa bullifera* (*n* = 26 with *C. laevis* colonies, *n* = 53 with *Azteca* sp. colonies).

Because colony size in Amazonian plant-ants is limited by the number of host-plant domatia [15], we used domatia number as a proxy for colony size. To estimate queen production as a function of colony size we destructively sampled 67 *Tococa bullifera* with *Crematogaster laevis*, *n* = 83 *T. bullifera* with *Azteca* sp., *n* = 87 *Maieta guienensis* with *C. laevis* and *n* = 101 *M. guianensis* with *Pheidole minutula*, all from nearby locations outside of the focal study area. Of these, 9, 9, 11, and 36 colonies (respectively), were reproductive. We used these reproductive colonies to estimate the relationship between colony size and queen production (Table 4); linear regression provided a better fit to the data than non-linear models (*results not shown*).

**Table 4:** Results of linear regressions testing for a relationship between the number of domatia a plant has and the number of queens counted in that a plant.

Ant species	Host plant	df	SS	SS	F value	P value		*R^2^*	Regression equation
			(regression)	(residual)					
*Crematogaster laevis*	*Maieta guianensis*	1,10	18.43	2.57	71.651	<0.0001	0.88	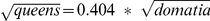	
*Pheidole minutula*	*Maieta guianensis*	1,35	228.54	40.46	197.72	<0.0001	0.85	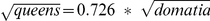	
*Azteca* sp.	*Tococa bullifera*	1,8	30.91	3.09	80.09	<0.0001	0.91	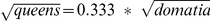	
*Crematogaster laevis*	*Tococa bullifera*	1,8	120.26	94.72	10.16	0.013	0.56	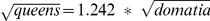	

Note that the intercept of all three regressions is zero because queens are only found in plants with at least one domatium.

We then established an array of greenhouse-grown seedlings in the center of the 9-ha plot (Figure 1). The array was composed of *n* = 50 *M. guianensis* (for colonization by *Pheidole minutula* or *Crematogaster laevis*) and *n* = 50 *T. bullifera* (for colonization by *Azteca* sp. or *C. laevis*). Seedlings had at least two fully expanded leaves with domatia and were arranged in a grid with species alternating and plants separated from each other by 10 m. Seedlings of *T. bullifera* were grown from seeds collected in Reserve 1501 and germinated in a shade house in moist sand; because of the difficulty in germinating *M. guianensis* seeds we collected vacant *M. guianensis* seedlings in the reserve and transplanted them to containers filled with local soil and maintained in the same shade-house. From July–September 2007 we surveyed the target seedlings 15, 35, and 90 days after transplanting to record the presence and species identity of queens. All queens found were removed to allow for subsequent colonization, which previous work has shown does not influence the probability of re-colonization [21]. There were *n* = 17 colonizations by *C. laevis*, *n* = 23 by *Azteca* sp., and *n* = 25 by *P. minutula*. *Crematogaster laevis* colonized *n* = 15 of its 100 potential host plant seedlings (15%), while *Pheidole minutula* colonized *n* = 17 out of 50 (34%) and *Azteca* sp. colonized *n* = 17 out of 50 (34%), while the remaining events were repeat colonizations of individual seedlings.

### Modeling framework

We used inverse models [26], [45] parameterized with the observational and experimental data described above to characterize the colonization of host plants by queens of our three focal species. This method assumes that observed spatial variation in colonization of host plants by queens is a multiplicative function of queen production, which is based on the size of potential queen sources (i.e., host-plant size), and local dispersal, which is modeled with a dispersal kernel that accounts for proximity of the sources to experimental host seedlings. For thorough reviews of inverse models and their construction, assumptions, and application see [24], [26], [45].

The total number of dispersing queens, *t*, produced by a colony was estimated as a linear function of the number of domatia its host plant has as follows:
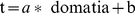
(1)where the parameter *a* determines the steepness in the increase in queen production with the number of domatia, and *b* determines the intercept of the domatia-queen production relationship.

We used a lognormal dispersal function, which considerable empirical and theoretical work has found to be the most appropriate function for a variety of dispersal mechanisms including animal movement ([46], [47], [48], [49], reviewed in 22). The kernel takes the form:
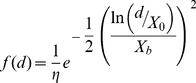
(2)


where *d* is the observed distance between the colony and the vacant host plant seedling, *X*_0_ is the distance at which maximum recruitment occurs (i.e., the mode of the dispersal kernel), *X*_b_ determines the breadth or spread of the dispersal kernel, and η is a normalization constant equal to the arcwise integration of the dispersal kernel [25].

Combining local queen production *Q* and the dispersal kernel results in a model for the potential number of queens in trap plant *i* over the course of our sampling interval:
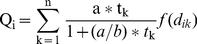
(3)where *t_k_* is the number of queens of *k* = 1…*n* colonies within the maximal dispersal distance (in meters) suggested by our model in the 9 ha plot, *d_ik_* is the distance from host plant *i* to source plant *k*, and *f()* is the lognormal dispersal kernel. For all analyses we assumed that the expected density of queens in a host plant follows a negative binomial distribution, reflecting the high degree of clumping observed in the data [50]. We used simulated annealing, a global optimization algorithm, to find the parameter values that maximized the likelihood of observed recruitment densities. We also calculated asymptotic 95% support limits for all the parameters. These Analyses were conducted using R v2.9.2 statistical software [51] and the packages “Likelihood 1.3” and “NeighLikeli 1.0”, as were all statistical analyses.
